# Adrenergic hormones induce extrapituitary prolactin gene expression in leukocytes-potential implications in obesity

**DOI:** 10.1038/s41598-018-20378-1

**Published:** 2018-01-31

**Authors:** Richard Barrett, Chandrakala Aluganti Narasimhulu, Sampath Parthasarathy

**Affiliations:** Burnett School of Biomedical Sciences, College of Medicine, University of Central Florida, Orlando, USA

## Abstract

The pituitary hormone prolactin (PRL), originally described for its role in lactation, has been implemented in over 300 functions and is produced by multiple cell types outside of the pituitary. Monocyte/macrophages in particular show robust expression of extra-pituitary prolactin (ePRL). While ePRL protein is identical to pituitary PRL and translated from the same gene, tissues outside the pituitary engage an alternative promoter to regulate expression. Many of the factors regulating this expression, however, remain unknown. Here we show that the adrenergic hormones epinephrine and norepinephrine induce PRL expression in the human monocytic cell line THP-1 at physiological concentrations. Furthermore, our experiments show the polarization state of differentiated macrophages can influence their response *in vitro*, with inflammatory M_1_ macrophages—common in obese adipose—showing the highest levels of PRL expression compared to other macrophage types. Adrenergic hormones have a clearly defined role in adipocyte lipid metabolism, stimulating lipolysis through hormone sensitive lipase (HSL) induction. Meanwhile, PRL has been shown to stimulate lipogenesis. This highlights ePRL production as a possible factor in obesity. The overall balance of these two signals could play a critical role in determining overall lipid turnover/accumulation in adipose depots where large numbers of adipose tissue macrophages (ATMs) reside.

## Introduction

PRL has been implemented in lipid metabolism ever since it’s discovered role in lactation and has been shown to induce lipogenesis in many tissues^1,2^ and inhibit lipolysis in adipocytes specifically^3^. More recent clinical studies have shown a correlation between hyperprolactinemia and weight gain in both males and females. Often, this weight gain is resolved when serum PRL levels are returned to normal^4,5^. The majority of these studies have been performed with pituitary-derived PRL in mind, but ePRL synthesis is prevalent in a variety of tissues across the human body^6,7^. Of particular interest to lipid metabolism is ePRL production in monocyte/macrophages^8^, cells that are numerous within adipose tissue. Such paracrine signaling from a local source of PRL—such as ATMs—could have far-reaching implications for the overall biological response of fat depots^9^.

Because ePRL is controlled by an alternative promoter to that or pituitary PRL, it is only within the last decade that researchers have begun to uncover some of the factors that regulate ePRL expression in monocyte/macrophages^10^. We propose that a class of molecules particularly relevant to lipid metabolism in adipocytes, the adrenergic hormones (AHs), may play a key role in regulating ePRL in monocytes/macrophages—more specifically in ATMs—and that the polarization state of the macrophages may have a further additive effect. As AH are well defined in stimulating lipolysis through activation of hormone sensitive lipase^11^, we propose such an ePRL response would oppose AH signaling on adipocytes.

**Figure 1:**
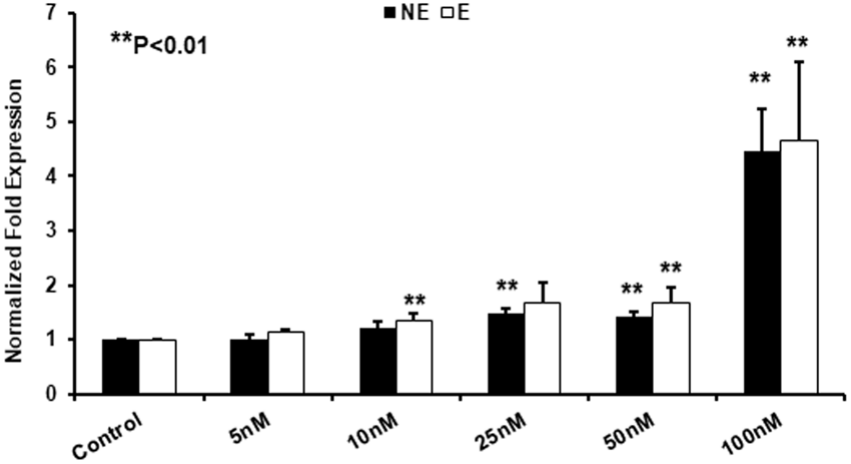
qRT-PCR of PRL gene expression in THP-1 monocytes after 24 hr treatment with norepinephrine or epinephrine.

In this study, we set out to determine the effects of dopamine and other catecholamines —such as the AH epinephrine (E) and norepinephrine (NE) —on ePRL in monocytes/macrophages. The effects of L-DOPA and dopamine produced no significant change in overall expression (data not shown); but upon exposure of cells to physiological concentration^12,13^ of norepinephrine and epinephrine, a dose dependent response occurred in the PRL gene. This response yielded a 4.5-fold increase in expression at 24hrs, at the maximal dose of 100 nM for norepinephrine, and a similar response in epinephrine (Fig. 1). AH exposure consistently increased PRL expression, but the degree to which gene expression was affected varied considerably between trials, with some individual trials yielding no significant change in expression at 100 nM, and others yielding an increase of over 40-fold at the same dose. We attempted to identify possible environmental factors influencing the response by altering conditions such as treatment time of day, confluence, and fetal bovine serum batches, none of which seemed to have a consistent effect on response magnitude. Further research needs to be done to identify other factors influencing the responses magnitude.

**Figure 2:**
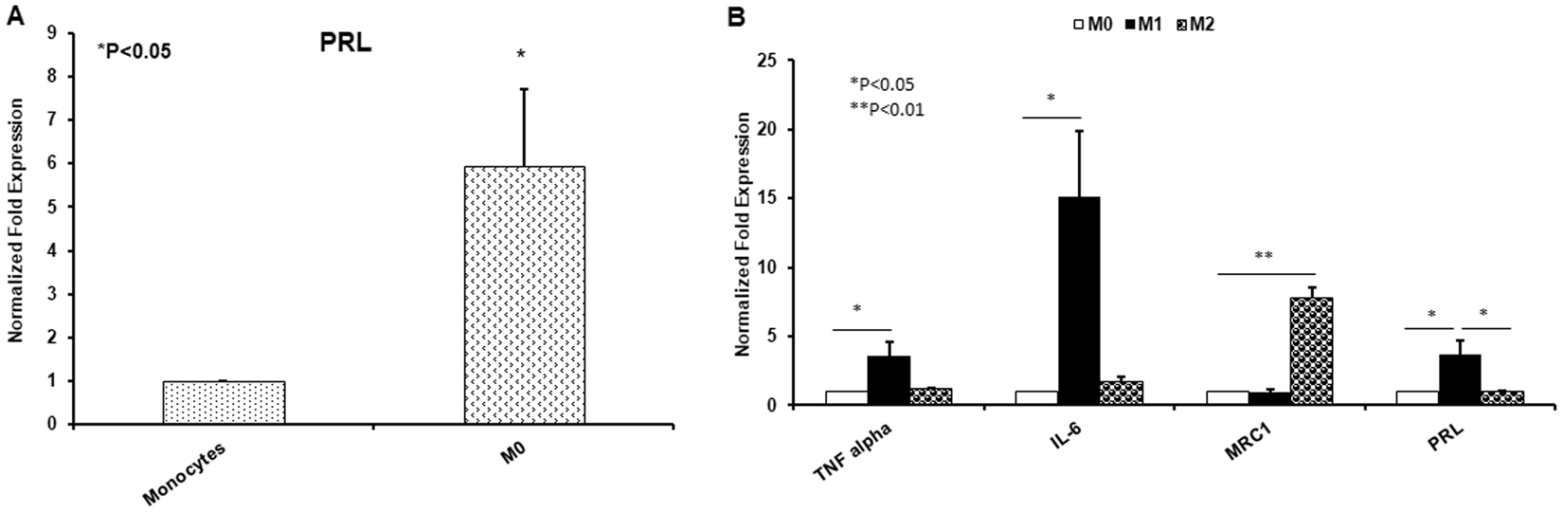
qRT-PCR of basal PRL gene expression in THP-1 monocytes and naïve M_0_ (**A**). qRT-PCR for polarization markers and PRL across polarization states.

Monocytes will typically leave the bloodstream and take up residence in various tissues as macrophages. Reports show that ATMs display different states of polarization depending on the health of an individual. The ATM population of obese subjects tends to display an M_1_ polarization and a greater overall number of ATMs to adipose cells. The ATMs of lean individuals, however, display an M_2_ polarization^14^. To simulate these populations *in vitro*, monocytes were exposed to phorbol 12-myristate 13-acetate (PMA) for 72 hours to make “naive” M_0_ macrophages. Naïve macrophages were then subjected to either lipopolysaccharide (LPS) for 24 hours to form the M_1_ population, or IL-4 for 24 hours to form the M_2_ population. Populations were verified *via* increases in common genetic markers (TNF alpha and IL6 for M_1_; MRC1 for M_2_) (Fig. 2B).

Before subjecting the populations to AH, we established a baseline for PRL gene expression in each cell type. Our results show a significant and consistent increase in PRL expression as monocytes differentiate into naive macrophages (Fig. 2A). This level of expression remains relatively unchanged as the macrophages polarize to M_2_. The progression of macrophages to the M_1_ state, however, is accompanied by a much larger increase in basal PRL gene expression (Fig. 2B).

**Figure 3:**
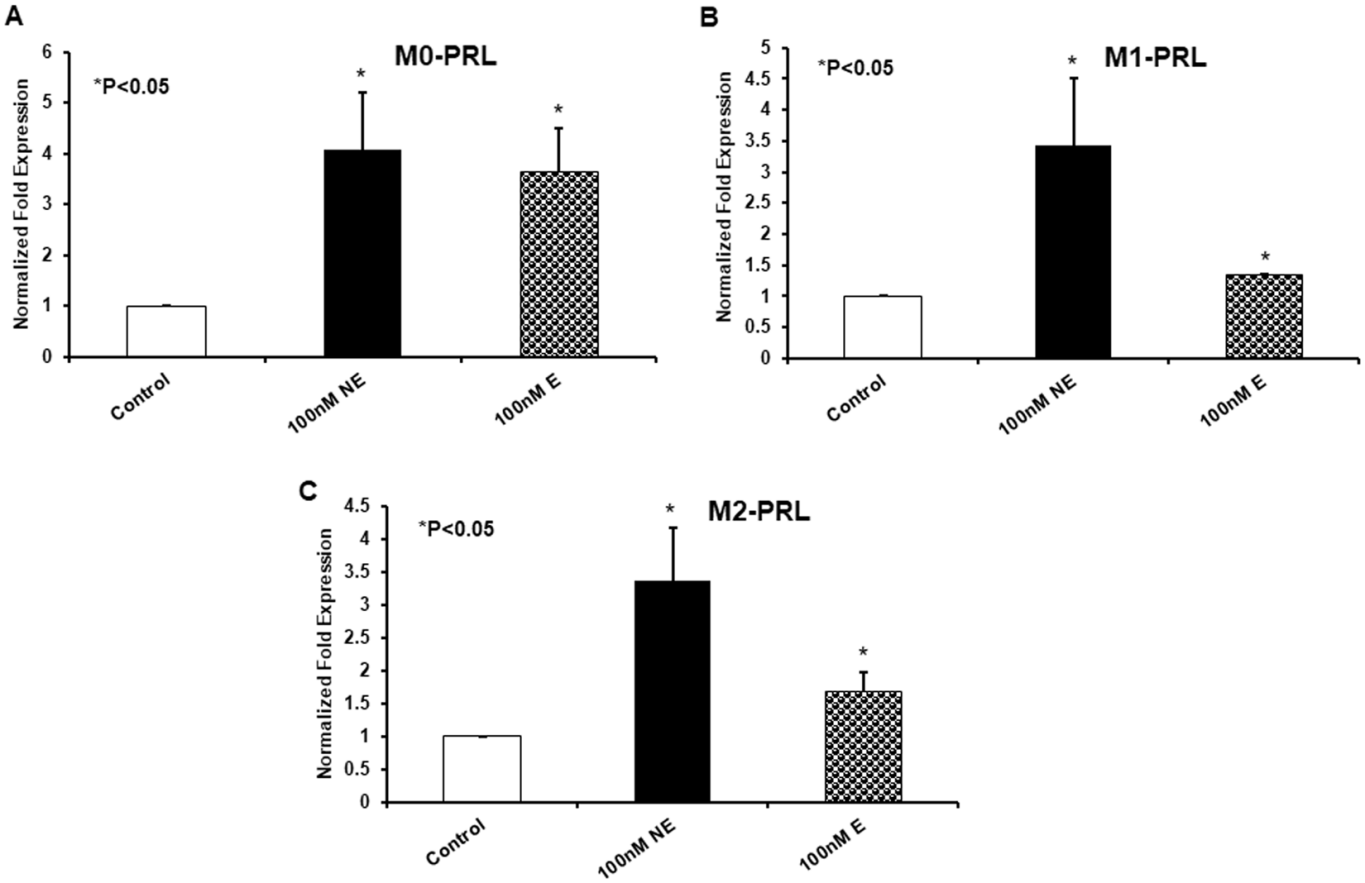
qRT-PCR of PRL gene expression after 24 hr treatment with norepinephrine or epinephrine in naïve M_0_ (**A**), inflammatory M_1_ (**B**), and anti-inflammatory M_2_ (**C**) monocyte derived macrophages.

Despite an already elevated basal expression of PRL in macrophages, norepinephrine treatment was still able to illicit an increase in PRL gene expression across all populations. Although M_1_ and M_2_ macrophages showed similar fold expression changes in the PRL gene upon AH exposure, overall expression in the M_1_ population was far higher as the fold expression was being compared to an already elevated basal level (Fig. 3B). Treatment with epinephrine yielded similar, but less drastic results on average when compared to norepinephrine (Fig. 3). The variability in the response noted in monocytes was far less pronounced in macrophages, likely because basal expression was already relatively high before treatment and thus not as susceptible to further increases.

Further work will be needed before the full physiological impact of this response can be understood. When considering the traditional roles of both players involved, this balance is likely to have wide-reaching impacts on cellular metabolism. Of particular interest are the opposing roles of the two signals. One of the primary functions of AH in the body is activating hormone sensitive lipase, which breaks down triglycerides to free fatty acids, moving them out of cells and into the bloodstream^11,15^. PRL, on the other hand, has been closely associated with increases in the body mass index (BMI), which is often corrected in hyperprolactinemia patients whose serum levels are returned to normal^5^.

With these findings in mind, it is easy to see how the interplay between these two cell signals residing in the adipose can have a strong influence on the overall state of the entire tissue. It is especially concerning considering that ATMs not only shift towards the M_1_ state in obese patients but also increase in number—accounting for up to 40% of the cells in an adipose depot^16^. Our findings that PRL expression increases as monocytes differentiate into macrophages could mean that such macrophage influxes could result in overall higher PRL levels, regardless of polarization state and could displace an already delicate balance of lipid mobilization and storage.

Another important aspect of this work is that ePRL production seems to be absent in most mammals. Previous studies have shown that in mice, rats, and most mammal species (outside of the primates), ePRL expression is virtually non-existent^10^. This was further verified in our lab using both mouse macrophage primary cells and cell lines, in all of which PRL gene expression was undetectable (data not shown). Thus, any impact of such signaling is an important divergence when translating metabolic and immunology research from animal models to humans. This could also help explain the high failure rates in the attempts to do so. Identifying and characterizing such pathways in general will be invaluable in bridging the gap for such translational research. Lastly, PRL has many other well-defined roles that act on immune cells directly, most notably being that of a co-mitogen^8^.

Seeing as the most well-defined regulator of traditional PRL expression in the pituitary is dopamine, which sharply inhibits its expression^17^ the paradoxical finding in this study that adrenergic hormones upregulated ePRL expression, necessitates further understanding how its expression is controlled at a local level, particularly in lieu of the involvement of inflammatory macrophages and obesity. Further research is needed to confirm if any of these proposed roles are performed by ePRL; but understanding the factors that regulate its expression is a key step in understanding any of its potential functions. Moreover, considering the already defined rolls of AH and PRL, it is not a stretch to conclude that their influence over each other could have far-reaching implications in general human physiology.

## Materials and Methods

### Reagents

RPMI-1640, FBS, penicillin-streptomycin, 1× PBS, TRIzol™, and primers were ordered from Life Technologies (Carlsbad, CA). Norepinephrine with bitartrate salt, epinephrine with bitartrate salt, phorbol 12-myristate 13-acetate (PMA), and lipopolysaccharide from *E. coli*, were ordered from Sigma-Aldrich (St. Louis, MO). IL-4 from Promega (Madison, WI), Sybr green, Superscript III cDNA kit from Invitrogen (Carlsbad, CA) were purchased and used.

### Cell Culture

The human monocytic cell line THP-1 was used to model both monocytes and macrophages. THP-1 monocytes were differentiated to “naive” macrophages with 50 ng/mL PMA for 72 hrs. The “naive” macrophages were then polarized to either inflammatory “M_1_” macrophages with 100 ng/mL LPS for 24 hrs or to anti-inflammatory “M_2_” macrophages with 20 ng/mL IL-4. Cells were maintained in RPMI-1640 supplemented with 10% FBS, 1% penicillin-streptomycin.

### Quantitative real-time PCR

Total RNA was isolated from cells using TRIzol™ reagent according to the manufacturer’s instructions; 1 µg of total RNA was used to synthesize cDNA, and quantitative real-time PCR was performed using the CFX96 Touch™ Real-Time PCR Detection System (Bio-Rad Laboratories, Hercules, CA) with SYBR Green as detection dye. mRNA levels of target genes were normalized to GAPDH.

### Statistical analysis

Values are presented as a mean ± standard deviation (SD), and statistical analyses were performed by using student’s t-test, with P < 0.05 as the level of significance.
